# The impact of nutritional, environmental, and lifestyle factors on neurological disorders: therapeutic implications and mechanistic insights

**DOI:** 10.3389/fphar.2026.1765786

**Published:** 2026-02-27

**Authors:** Dib Chakif, Julien Furrer

**Affiliations:** Department of Chemistry, Biochemistry and Pharmaceutical Sciences, University of Bern, Bern, Switzerland

**Keywords:** environment, epigenetics, gut-brain axis, lifestyle, mitochondria, neuroinflammation, neurology, nutrition

## Abstract

Neurological disorders like Alzheimer’s, Parkinson’s, multiple sclerosis, and primary psychiatric conditions are complex, arising from a mix of genetic and modifiable risks. Growing evidence indicates that nutrition, environment, and lifestyle significantly influence disease development, progression, and treatment response. Nutrients such as vitamins, minerals, omega-3 fatty acids, and polyphenols affect neuroinflammation, oxidative stress, mitochondrial health, and neurotransmitter function. Dietary patterns like the Mediterranean and ketogenic diets offer protective benefits in clinical and experimental contexts. Meanwhile, environmental neurotoxicants–air pollution, heavy metals, pesticides, and endocrine disruptors contribute to neurodegeneration via oxidative damage, synaptic impairment, and epigenetic alterations. Lifestyle factors, such as physical activity, sleep, stress, and substance use, affect brain plasticity, neurogenesis, and metabolic health, thereby influencing disease progression over time. These factors often share common pathways such as oxidative stress, inflammation, vascular injury, mitochondrial dysfunction, and protein misfolding, underscoring the need for a comprehensive prevention and treatment strategy. Emerging therapies now incorporate personalized nutrition, lifestyle changes, and environmental risk mitigation alongside traditional drugs, supported by advances in multi-omics, digital health, and systems biology. Public health efforts to reduce neurotoxic exposure and encourage healthy habits further strengthen these approaches. This review summarizes existing mechanistic and clinical knowledge, with a focus on the potential of nutritional, environmental, and lifestyle interventions in neurological diseases. It also outlines the future research required to enhance precision neurology and strategies for brain health prevention.

## 1 Introduction

Neurological disorders are a significant and growing global health issue, affecting millions of people across all ages (WHO, 2022). Conditions such as Alzheimer’s, Parkinson’s, multiple sclerosis, epilepsy, stroke, and neurodevelopmental disorders result in high rates of illness and death, and they impose a significant socioeconomic burden on healthcare systems worldwide (Ascherio and Schwarzschild, 2016; Livingston et al., 2020). The Global Burden of Disease (GBD) study reveals that these disorders cause more disability-adjusted life years (DALYs) than nearly any other group of diseases, underscoring their widespread prevalence and long-lasting impact on the quality of life (Feigin et al., 2020). In addition to neurodegenerative and neurodevelopmental conditions, psychiatric disorders (e.g., depression, anxiety, and schizophrenia) are also influenced by modifiable nutritional, environmental, and lifestyle factors, with shared pathways involving inflammation, metabolic–vascular health, and stress biology.

Despite notable progress in neuroscience and pharmacology, we still lack effective treatments for many neurological disorders. Existing therapies mainly address symptoms rather than altering the disease course, highlighting the urgent need for new preventive and therapeutic approaches. Growing evidence shows that modifiable factors such as nutrition, environmental exposures, and lifestyle habits significantly affect neurological health (Lahoda Brodska et al., 2023). These factors interact with genetic predispositions and molecular pathways, influencing the development, progression, and severity of neurological conditions.

Nutritional status is vital for brain health throughout life. Deficiencies in nutrients such as B12, folate, vitamin D, and magnesium are associated with cognitive decline and neuropsychiatric problems (Tardy et al., 2020; Chen et al., 2024; Rotstein et al., 2022). Conversely, neuroprotective diets like the Mediterranean and ketogenic diets can help manage conditions from Alzheimer’s to epilepsy. Environmental factors, including air pollution, pesticides, heavy metals, and endocrine disruptors, also play a significant role in neurodegeneration and developmental disorders (Landrigan et al., 2018).

Additionally, lifestyle habits such as regular exercise, quality sleep, stress management, and avoiding harmful substances are essential for neuronal plasticity, reducing inflammation, and enhancing resilience (Xie et al., 2013). These influences operate across the life course from prenatal brain development and childhood neuroplasticity to midlife risk accumulation and late-life vulnerability–supporting a life-course model of brain health and disease risk.

Importantly, these domains are interconnected rather than isolated, sharing mechanistic pathways like oxidative stress, mitochondrial dysfunction, neuroinflammation, and epigenetic reprogramming (Barnham et al., 2004). The gut-brain axis highlights the link between diet, microbiome, immune responses, and environmental and lifestyle factors in influencing neurological health (Cryan et al., 2019). Recognizing these interconnected mechanisms allows researchers and clinicians to design comprehensive therapies that go beyond traditional single-target drug approaches (Barabási et al., 2011; Ngandu et al., 2015). Unlike prior reviews that focus on single modifiable domains, we synthesize nutrition, environmental exposures, and lifestyle behaviors within a unified, multidomain framework, linking convergent mechanisms to clinical prevention/adjunctive care and to population-level public health and policy actions.

This review consolidates current evidence on how nutritional, environmental, and lifestyle factors affect neurological disorders, emphasizing mechanistic insights and therapeutic possibilities. Our objectives are to (i) critically evaluate dietary and nutritional approaches for neurological health, (ii) explore how environmental toxins and pollutants contribute to disease development, (iii) assess the influence of lifestyle choices on brain function, (iv) uncover the mechanisms connecting these factors, and (v) outline emerging treatments and future research avenues. By offering a comprehensive, multidisciplinary overview, this review seeks to improve understanding of prevention and treatment strategies for neurological disorders within the context of precision medicine. Throughout, we highlight how mechanistic insights can inform practical steps in clinical settings (dietary/lifestyle assessment and counseling, exposure history-taking, and risk factor modification), as well as preventive strategies deliverable through primary care and public health programs.

## 2 Nutritional factors and neurological disorders

### 2.1 Micronutrients and brain health

Ensuring sufficient intake of micronutrients is essential for healthy neuronal growth, neurotransmission, and safeguarding against neurodegenerative conditions (see Table 1). Lack of specific vitamins and minerals is strongly associated with cognitive decline, mood issues, and neurodegenerative diseases (Lahoda Brodska et al., 2023).

**TABLE 1:** Micronutrients with essential neurological roles, highlighting their mechanisms, clinical implications of deficiency or excess, and primary dietary sources.

Micronutrient	Core neurological roles	Deficiency/Excess signals	Mechanisms (high level)	Major dietary sources	Refs
Vitamin B12	Myelination; one-carbon metabolism; neurotransmission	↑ Homocysteine; cognitive decline; neuropathy	DNA methylation; myelin integrity; homocysteine remethylation	Animal products; fortified foods	Smith et al. (2018), Kennedy (2016)
Folate (B9)	One-carbon metabolism; neurodevelopment	↑ Homocysteine; neural tube defects	DNA methylation; nucleotide synthesis	Leafy greens; legumes; fortified grains	-
Vitamin B6	Neurotransmitter synthesis (GABA, serotonin, dopamine)	Seizures; mood symptoms; ↑ homocysteine	Cofactor in amino acid metabolism; homocysteine control	Poultry; fish; potatoes; bananas	-
Vitamin D	Neurosteroid; synaptic plasticity; immune modulation	Cognitive impairment; MS signals	Calcium homeostasis; anti-oxidative and anti-inflammatory signaling	Sunlight; fatty fish; fortified dairy	Sintzel et al. (2018)
Vitamin E	Lipid antioxidant; membrane protection	Accelerated neurodegeneration (deficiency)	Limits lipid peroxidation; preserves PUFA membranes	Nuts; seeds; vegetable oils	-
Vitamin C	Antioxidant; neurotransmitter synthesis	Fatigue; mood/cognitive issues	Regenerates vitamin E; dopamine→NE cofactor	Citrus; berries; peppers	-
Magnesium	NMDA modulation; neuronal excitability	Depression; epilepsy signals	Antagonizes excitotoxicity; ATP enzyme support	Whole grains; nuts; legumes; greens	Chen et al. (2024)
Zinc	Synaptic plasticity; enzyme function	Dysregulated homeostasis ↔ amyloid pathology	Modulates NMDA/AMPA; metalloprotease activity	Oysters; red meat; legumes; nuts	Kozin (2023)
Iron	Myelination; mitochondrial enzymes; dopamine synthesis	Deficiency: cognitive/motor issues; excess: oxidative damage	Tyrosine hydroxylase cofactor; Fenton chemistry	Red meat; legumes; fortified grains	-
Selenium	Selenoproteins (GPx, TrxR); antioxidant defense	Reduced neuronal resilience (low status)	Reduces oxidative stress; thyroid hormone activation	Brazil nuts; seafood; eggs	Solovyev (2015)

B vitamins are crucial for brain health. In particular, vitamin B12 and folate are key players in one-carbon metabolism, influencing DNA methylation, nerve myelination, and homocysteine levels. Elevated homocysteine, often due to B12, folate, or B6 deficiencies, is associated with higher risks of Alzheimer’s, vascular dementia, and stroke (Moore et al., 2012). During pregnancy, inadequate folate intake markedly increases the risk of neural tube defects, highlighting the importance of proper prenatal nutrition for healthy neurodevelopment (WHO, 2025). Choline is an essential nutrient required for acetylcholine synthesis and membrane phospholipid metabolism, and prenatal choline availability can influence neurodevelopment and later-life cognitive trajectories (Paules et al., 2025; Mujica-Coopman et al., 2024). Iodine is likewise critical in early life because thyroid hormones are indispensable for fetal brain development; even mild-to-moderate iodine deficiency during pregnancy has been associated with poorer neurodevelopmental outcomes (De Escobar et al., 2007; Grossklaus et al., 2023).

Vitamin D is now recognized as a neurosteroid that affects neurogenesis, synaptic plasticity, and immune response. Low levels of vitamin D in the blood are linked to higher risks of multiple sclerosis, Parkinson’s disease, and cognitive decline. Mechanistically, vitamin D helps regulate calcium levels, reduces oxidative stress, and influences inflammation pathways (Sintzel et al., 2018).

Vitamin E, a potent lipid-soluble antioxidant, helps protect neuronal membranes from oxidative damage. A deficiency speeds up neurodegeneration and is linked to Alzheimer’s disease progression. Likewise, vitamin C is involved in neurotransmitter synthesis and helps combat oxidative stress (Ulatowski and Manor, 2015).

Minerals like magnesium, zinc, iron, and selenium are essential for maintaining brain health. A magnesium deficiency can interfere with NMDA receptor regulation, leading to increased excitotoxicity, which is linked to depression and epilepsy (Chen et al., 2024). Zinc is essential for synaptic plasticity, but abnormal levels may encourage amyloid buildup in Alzheimer’s disease (Kozin, 2023). Iron is necessary for dopamine production; however, too much iron can cause oxidative damage and is associated with a higher risk of Parkinson’s disease. Selenium, as part of antioxidant selenoproteins, protects neurons from oxidative stress (Solovyev, 2015). Clinical note: supplementation is not risk-free–excess intake of fat-soluble vitamins (A, D, E, K) may accumulate and cause toxicity, while high-dose iron or selenium can be harmful; supplementation should therefore be individualized (ideally guided by dietary assessment and laboratory evaluation) rather than assumed to be universally beneficial (Tardy et al., 2020).

Together, these micronutrients demonstrate how even minor dietary deficiencies can have a profound impact on neurological health. Table 1 summarizes core roles, clinical signals, and representative sources.

Nutritional vulnerabilities also differ by life stage: during prenatal/early development, micronutrient adequacy (e.g., iodine and choline) supports neurogenesis and myelination; in adulthood, diet quality interacts with cardiometabolic and inflammatory risk; and in ageing, reduced intake/absorption and multimorbidity can increase susceptibility to deficiencies and to adverse effects from unnecessary high-dose supplements.

### 2.2 Macronutrients and dietary patterns

Besides specific nutrients, overall dietary habits influence brain health via complex metabolic and inflammatory pathways. Western-style diets high in fat and sugar (see Table 2) are commonly associated with diminished cognitive abilities, less neurogenesis in the hippocampus, and a greater dementia risk (Kanoski and Davidson, 2011). Consistently, consuming these foods can cause insulin resistance, neuroinflammation, and gut microbiota alterations, all of which promote neurodegeneration.

**TABLE 2:** Summary of major dietary patterns and bioactive food compounds with relevance to brain health, outlining their characteristic features, neurological outcomes, underlying mechanisms, practical considerations, and supporting evidence.

Diet/Pattern	Defining features	Neurological outcomes (direction)	Dominant mechanisms	Caveats/Notes	Refs
Western/high-fat–high sugar	Energy-dense, refined carbs, saturated/trans fats, low fiber	↓ cognition, ↓ hippocampal neurogenesis, ↑ dementia risk	Insulin resistance, neuroinflammation, dysbiosis	Confounding lifestyle factors are common	Kanoski and Davidson (2011)
Mediterranean (MD)	Fruits/veg, whole grains, legumes, olive oil, fish, nuts; moderate wine	Slower cognitive decline; ↓ AD risk	Anti-inflammatory, antioxidant, MUFA/PUFA profile, vascular support	Adherence varies by cohort	Vlachos and Scarmeas (2019)
Ketogenic (KD)	Very low carb, high fat → nutritional ketosis	Anti-seizure efficacy; emerging benefits in PD/AD	↑ mitochondrial efficiency; ↓ excitotoxicity; ketone signaling	enhance dietary strategies tailored to each individual’s	Gano et al. (2014)
Plant-based (incl. vegan)	High fiber, polyphenols; low animal products	↓ neuroinflammation; improved vascular health; potential ↓ stroke/dementia risk	Polyphenols, SCFAs via microbiota, improved metabolic profile	Monitor B12, iron, iodine, omega-3 (EPA/DHA)	Kobayashi et al. (2020)
DASH/MIND variants	Veg/fruit emphasis, low sodium, whole grains, low red/processed meat	↓ dementia and stroke risk	Vascular and BP control; antioxidant load	MIND blends MD + DASH for cognition	Nucci et al. (2024)
Compound /class	Food sources	Primary neurological effects	Mechanistic highlights	Evidence signals	​
Flavonoids (quercetin, epicatechin)	Berries, tea, cocoa, apples	↑ BDNF, improved memory/executive function; anti-amyloid	Antioxidant; anti-inflammatory; synaptic plasticity signaling	Human cohorts + RCTs on cognition in aging	Ayaz et al. (2019)
Resveratrol (stilbene)	Grapes, red wine, peanuts	Neuroprotection; possible cognitive benefits	Sirtuin activation; anti-inflammatory; vascular	Dose/bioavailability constraints	Taylor and Holscher (2020)
Omega-3 PUFA (DHA/EPA)	Fatty fish, algae oils	Membrane fluidity, improved cognition; antidepressant adjunct	Resolvin signaling; anti-inflammatory; synaptogenesis	The benefits are stronger with a higher DHA status	Calder (2015)
Curcumin	Turmeric	Anti-amyloid; anti-inflammatory; antioxidative	Crosses BBB; modulates NF-κB/oxidative pathways	Bioavailability formulations vary	Hamaguchi et al. (2010)
Caffeine	Coffee, tea	↑ alertness; possible long-term neuroprotection	Adenosine receptor antagonist; ↑ neurotransmission	Sensitivity, sleep, and anxiety considerations	López-Cruz et al. (2018)
Theobromine	Cocoa	Mild CNS stimulant; mood/cognition support	Adenosine modulation: vascular effects	Lower potency vs. caffeine	Jacobs et al. (2022)

Evidence for dietary patterns comes from multiple streams: observational cohorts linking habitual patterns to long-term cognitive outcomes, randomized or quasi-experimental dietary interventions assessing intermediate endpoints, and experimental/preclinical studies probing mechanistic plausibility; interpreting claims requires attention to this hierarchy of evidence.

On the other hand, the Mediterranean diet (MD). A diet that includes fruits, vegetables, whole grains, olive oil, fish, and nuts has been associated with slower cognitive decline and a reduced risk of Alzheimer’s disease. The anti-inflammatory and antioxidant properties of MD, along with its beneficial fatty acid profile, are thought to provide neuroprotective benefits (Nucci et al., 2024; Vlachos and Scarmeas, 2019).

The ketogenic diet (KD), which is high in fat and protein and very low in carbohydrates, induces ketosis and has been used for decades to treat refractory epilepsy. Recent evidence indicates that KD might also benefit neurodegenerative diseases by improving mitochondrial function, decreasing excitotoxicity, and boosting neuronal energy metabolism (Gano et al., 2014).

Plant-based diets rich in polyphenol-containing foods are linked to decreased neuroinflammation and enhanced vascular health, potentially reducing the risk of stroke and dementia (Tayab et al., 2022). However, strict vegan diets should ensure adequate intake of nutrients such as vitamin B12, iron, and omega-3 fatty acids to prevent neurological deficiencies (Ursea et al., 1975).

### 2.3 Bioactive compounds and phytochemicals

Certain bioactive compounds exhibit significant neuromodulator effects regardless of their caloric or macronutrient content (see Table 2). Polyphenols, found abundantly in berries, tea, coffee, and cocoa, are known for their strong antioxidant and anti-inflammatory properties. For polyphenols and omega-3 fatty acids, human evidence is strongest for observational associations and select randomized supplementation trials on intermediate outcomes, while many neuroprotective mechanisms (antioxidant signaling, microglial modulation, synaptic effects) remain largely supported by experimental and preclinical research; this should temper causal interpretation.

Flavonoids such as quercetin, epicatechin, and resveratrol enhance synaptic plasticity, boost BDNF (brain-derived neurotrophic factor) levels, and provide protection against amyloid pathology (Khomenko et al., 2025; Ge et al., 2011).

Omega-3 fatty acids, particularly docosahexaenoic acid (DHA), are crucial for maintaining neuronal membrane fluidity and supporting neurotransmission. Supplementing with DHA has shown positive effects on cognitive function and depression, while deficiencies are associated with increased risks of Alzheimer’s disease and mood disorders (Tanaka et al., 2012).

Curcumin, which is derived from turmeric, has attracted interest because it can pass through the blood–brain barrier and affect various pathways such as amyloid aggregation, oxidative stress, and neuroinflammation (Yang et al., 2005).

Caffeine and theobromine, mild central nervous system stimulants present in coffee and cocoa, boost alertness and might offer long-term neuroprotection by modulating adenosine receptors (EFSA Panel on Dietetic Products and Nutrition and Allergies NDA, 2014; MartÃ-nez-Pinilla et al., 2015).

### 2.4 Mechanistic insights

The beneficial impact of nutrition on neurological disorders involves several shared mechanisms. Antioxidants such as vitamins E and C, polyphenols, and selenium help control oxidative stress by neutralizing free radicals and preventing lipid peroxidation. Addressing neuroinflammation involves using omega-3 fatty acids, vitamin D, and polyphenols. Mitochondrial health can be supported by ketogenic diets and specific micronutrients that enhance energy metabolism and reduce excitotoxicity.

Neurotransmitter synthesis relies on cofactors such as B vitamins, iron, and vitamin C, which are essential for pathways that generate serotonin, dopamine, and acetylcholine (Al Mughram et al., 2022).

Epigenetic regulation involves nutrients such as folate, B12, and polyphenols that influence DNA methylation and histone modifications, thereby affecting gene expression in neuronal cells (Crider et al., 2012). Regarding the modulation of the gut–brain axis: Dietary fibres and polyphenols impact microbiota composition, resulting in the production of specific metabolites (Filosa et al., 2018).

Microbiome-mediated effects are partly conveyed by neuroactive metabolites, including short-chain fatty acids (e.g., acetate, propionate, butyrate) that can influence microglial maturation and blood–brain barrier integrity, and tryptophan-derived metabolites (e.g., indoles and kynurenine-pathway products) that modulate immune signaling and neurotransmission (Fock and Parnova, 2023).

### 2.5 Therapeutic implications

Nutritional interventions are increasingly acknowledged as supplementary options for neurological conditions. Clinical trials, also referenced in Table 3, indicate that B-vitamin supplements reduce homocysteine levels and slow brain atrophy in individuals with mild cognitive impairment (Smith et al., 2018). Vitamin D supplements have been shown to alleviate fatigue and decrease relapse rates in multiple sclerosis. The ketogenic diet remains the preferred treatment for drug-resistant epilepsy, and recent studies suggest it may also offer benefits for Parkinson’s and Alzheimer’s diseases (Paul et al., 2017).

**TABLE 3:** Dietary and supplement-based interventions for neurological health, detailing target groups, observed clinical benefits, and key practical considerations.

Intervention	Target population/Condition	Clinical signal	Practical considerations	Refs
B-vitamin supplementation (B6, B9, B12)	Mild cognitive impairment; hyperhomocysteinemia	↓ homocysteine; slowed brain atrophy; cognitive stabilization	Check baseline B12/folate; avoid masking B12 deficiency	Smith et al. (2018)
Vitamin D supplementation	Multiple sclerosis; low 25(OH)D	Improved fatigue; ↓ relapse/activity signals in some trials	Dose to target serum 25(OH)D; monitor calcium	Munger et al. (2006)
Ketogenic diet (classic, MCT, modified Atkins)	Drug-resistant epilepsy; exploratory in PD/AD	Gold-standard efficacy in refractory epilepsy; emerging neurodegenerative data	Medical supervision; micronutrient sufficiency; adherence	Paoli et al. (2014)
Mediterranean MIND/DASH patterns	General/at-risk older adults	↓ dementia and stroke risk; slower cognitive decline	Emphasize adherence, cultural fit, and affordability	Vlachos and Scarmeas (2019)
Polyphenol-rich dietary strategies	Aging populations; metabolic risk	Improved memory/executive function	Whole-food focus; bioavailability varies by matrix	Spencer (2009)
Omega-3 (DHA/EPA)	Cognitive impairment, depression adjunct	Cognitive and mood benefits in subsets	Aim for adequate DHA; consider algae oils for vegans	Bazinet and Layé (2014)

The Mediterranean and DASH (Dietary Approaches to Stop Hypertension) diets are associated with a reduced risk of dementia and stroke. Eating diets rich in polyphenols can improve memory and executive functions in older adults. While dietary interventions do not replace pharmacological treatments, they provide a valuable, affordable, and accessible way to support prevention. Personalized nutrition, guided by genomics and metabolomics, can improve dietary strategies customized to everyone’s risk profile (Nucci et al., 2024).

## 3 Environmental factors and neurological disorders

Environmental exposures are increasingly recognized as essential influences on neurological health (Landrigan et al., 2018). Unlike genetic factors, modifiable factors are vital targets for prevention and intervention. Growing evidence from epidemiological and mechanistic studies connects air pollution, heavy metals, pesticides, and endocrine-disrupting chemicals to the onset and advancement of neurological disorders (Althomali et al., 2024).

An exposome perspective is also increasingly used to reflect real-world conditions in which individuals experience mixtures of low-dose, chronic exposures across the lifespan; mixture effects may be non-additive and can intersect with nutrition, lifestyle, and socioeconomic context (Dallere et al., 2025).

Beyond well-studied toxicants (air pollution, metals, pesticides, endocrine disruptors), emerging exposures attracting increasing attention include micro- and nanoplastics, per- and polyfluoroalkyl substances (PFAS), noise pollution, and artificial light at night, all of which may interact with stress, sleep, and metabolic pathways relevant to brain health (Ames et al., 2025; Ma et al., 2025).

### 3.1 Air pollution and neurodegeneration

Air pollution is now recognized not only as a threat to the heart and lungs but also as a significant neurotoxic danger. Delicate particulate matter (PM2.5), nitrogen dioxide (NO_2_), ozone, and ultrafine particles can infiltrate the respiratory system, reach the bloodstream, and cross the blood–brain barrier (BBB) (see Table 4) (Oberdörster et al., 2004; Xu et al., 2023).

**TABLE 4:** Air pollutants linked to neurodegeneration, proposed mechanisms, and exemplar metrics.

Pollutant/Type	Exposure route/Metric	Neurological outcomes	Dominant mechanisms	Refs
PM2.5 (delicate particulate matter)	Ambient air; annual/long-term μg/m^3^	↑ Alzheimer’s and Parkinson’s risk; accelerated cognitive decline; early amyloid/tau pathology in youth	Systemic/neuronal oxidative stress; microglial activation; vascular dysfunction; olfactory translocation; BBB disruption	Elder et al. (2006)
Ultrafine particles (UFPs)	Ambient air; number concentration; high near traffic	Cognitive impairment; synaptic alterations	Direct CNS entry via olfactory nerve; mitochondrial damage; inflammation	Xu et al. (2023)
NO_2_	Traffic-related; ppb	↓ cognitive performance; neuroinflammation signals	Oxidative stress; endothelial dysfunction; nitrative stress	-
Ozone (O_3_)	Ambient air; ppb (8-h max)	Associations with cognitive decline and dementia in some cohorts	Lipid peroxidation; systemic inflammation impacting the CNS	-

Epidemiological studies link chronic exposure to PM2.5 with a higher risk of Alzheimer’s disease, Parkinson’s disease, and faster cognitive decline in aging populations. Autopsy studies have found amyloid plaques and tau pathology in children and young adults living in highly polluted urban areas, indicating that air pollution may trigger neurodegenerative processes decades before clinical symptoms appear (Calderón-Garcidueñas et al., 2012).

Mechanistically, inhaled particulates induce systemic inflammation, oxidative stress, and vascular dysfunction, which later impact the CNS. Ultrafine particles may directly translocate to the brain through the olfactory pathway, bypassing the BBB. These exposures disrupt microglial homeostasis, promote amyloid aggregation, and impair synaptic integrity (Elder et al., 2006).

### 3.2 Heavy metals and neurological toxicity

Heavy metals such as lead, mercury, cadmium, and arsenic are persistent pollutants in the environment that are well-known for their neurotoxic effects, as explained in Table 5 (Jaishankar et al., 2014).

**TABLE 5:** Persistent metals, exposure pathways, and neurotoxic profiles.

Metal	Major sources/Exposure	Neurological effects	Mechanistic highlights	Life-stage vulnerability	Refs
Lead (Pb)	Legacy paint, contaminated dust/soil, plumbing, and occupational	↓ IQ, attention deficits, behavioral disorders; adult: cognitive decline	Calcium mimicry; impaired synaptic pruning; neurotransmitter dysregulation	High in prenatal/childhood exposure	Balali-Mood et al. (2021)
Methylmercury (MeHg)	Seafood (bioaccumulation), industrial releases	Motor dysfunction, cognitive impairment, and visual field deficits	Mitochondrial dysfunction; oxidative stress; microtubule disruption	Prenatal/early life is highly susceptible	-
Arsenic (As)	Contaminated groundwater, rice, and industrial	Memory deficits; ↑ neurodegeneration risk	DNA damage; epigenetic modifications; altered neurogenesis	All ages; prenatal sensitivity is notable	-
Cadmium (Cd)	Tobacco smoke, industrial emissions, and certain foods	Olfactory dysfunction; dopaminergic neurotoxicity (PD relevance)	Oxidative stress; metal–protein interactions	Cumulative with age	-

Lead exposure, especially during childhood, is linked to lower IQ, attention issues, and a higher risk of behavioural disorders (Lanphear et al., 2005). It disrupts synaptic pruning, calcium signalling, and neurotransmitter release (Bressler and Goldstein, 1991). Mercury, particularly as methylmercury, accumulates in seafood and leads to motor impairments, cognitive difficulties, and visual disturbances. It disrupts mitochondrial activity and increases oxidative stress within neurons (Novo et al., 2021). Exposure to arsenic through contaminated drinking water has been linked to memory problems and a higher risk of neurodegenerative diseases. It causes DNA damage, epigenetic changes, and interference with neurogenesis (Tyler and Allan, 2014; Benskey et al., 2012).

Although less researched, cadmium is linked to olfactory dysfunction and dopaminergic neurotoxicity, which are both relevant to Parkinson’s disease (Raj et al., 2021). These metals tend to bioaccumulate and cause prolonged neurotoxic effects, even with low exposure levels (Balali-Mood et al., 2021).

### 3.3 Pesticides and Parkinson’s disease

Epidemiological evidence strongly connects chronic pesticide exposure to Parkinson’s disease (PD). Certain compounds, such as paraquat, rotenone, and organophosphates, have been shown to cause selective dopaminergic neurotoxicity (Tanner et al., 2011).

Mechanistic studies (see Table 6) have shown that these pesticides impair mitochondrial complex I, increase reactive oxygen species (ROS), and activate pro-inflammatory pathways, thereby mimicking key features of PD pathology (Sherer et al., 2003). Genetic susceptibility (for example, polymorphisms in detoxification enzymes like GSTs or PON1) may further increase vulnerability to pesticide-induced neurotoxicity (Paul et al., 2017; (Menegon et al., 1998).

**TABLE 6:** Representative pesticides implicated in PD risk and shared mechanisms.

Pesticide/Class	Evidence signals (epi/toxicology)	Mechanistic convergence	Genetic modifiers	Typical exposure contexts	Refs
Paraquat (herbicide)	Strong epidemiological links to PD; animal models show nigrostriatal loss	↑ ROS; redox cycling; mitochondrial injury	GST/PON1 variants may heighten risk	Agricultural application; drift; occupational handling	-
Rotenone (insecticide)	Associations with PD: reproduces PD-like pathology in rodents	Complex I inhibition; α-syn aggregation; neuroinflammation	Mitochondrial gene variants; detox enzyme polymorphisms	Agricultural/pest control	-
Organophosphates (e.g., chlorpyrifos)	Cohort/case–control links; cholinesterase inhibition markers	Mitochondrial stress; neuroinflammation	PON1 polymorphisms	Agriculture; domestic use (legacy)	-

More broadly, gene–environment interactions (including detoxification enzymes, oxidative stress responses, and inflammatory signaling) represent a cross-cutting theme that supports a precision-neurology approach to risk stratification and targeted prevention.

### 3.4 Endocrine disruptors and neurodevelopment

Endocrine-disrupting chemicals (EDCs), such as bisphenol A (BPA), phthalates, and polychlorinated biphenyls (PCBs) (see Table 7), disrupt hormonal signaling pathways crucial for brain development and function (Gore et al., 2015). Because endocrine signaling and epigenetic programming are central during development, environmental exposures in critical windows may have long-lasting, and potentially transgenerational effects through heritable epigenetic alterations observed in animal models and increasingly explored in human epigenomic studies (Klibaner-Schiff et al., 2024).

**TABLE 7:** EDC exposures linked to neurodevelopmental outcomes and mechanisms.

EDC/Class	Common sources	Neurodevelopmental outcomes	Mechanistic pathways	Windows of susceptibility	Refs
Bisphenol A (BPA)	Polycarbonate plastics, can linings, thermal paper	↑ ASD/ADHD risk signals; cognitive/behavioral changes	Estrogen receptor mimicry; neuroendocrine disruption; epigenetic reprogramming	Prenatal and early childhood	Gore et al. (2015)
Phthalates	Personal care products, PVC plastics, and medical tubing	Attention/executive function deficits; ASD/ADHD associations	Androgen/thyroid disruption; synaptic/connectivity changes; epigenetics	Prenatal/early life	-
PCBs (legacy POPs)	Legacy electrical fluids, contaminated fish/soil/dust	Cognitive deficits; developmental delay	Thyroid hormone interference; oxidative stress; epigenetic modifications	Prenatal/infancy; lactational transfer	-

Prenatal and early-life exposure to endocrine-disrupting chemicals (EDCs) is associated with an increased risk of autism spectrum disorders (ASD), attention-deficit/hyperactivity disorder (ADHD), and cognitive impairments (Özel and Rüegg, 2023). For instance, BPA mimics estrogen, impacting neuroendocrine signaling, synaptic connections, and gene expression in the developing brain. Similarly, PCBs disrupt thyroid hormone regulation, which is crucial for proper neurodevelopment (Chung et al., 2011; Gilbert et al., 2020).

These compounds often work through epigenetic reprogramming, altering DNA methylation and histone modifications in neural tissues, resulting in long-lasting effects even after exposure ceases (Alavian‐Ghavanini and Rüegg, 2018).

### 3.5 Mechanistic insights

Environmental exposures' neurotoxic effects affect multiple mechanistic pathways (see Table 8). Oxidative stress and mitochondrial dysfunction: Air pollutants, heavy metals, and pesticides cause ROS production and damage to mitochondria, leading to neuronal apoptosis.

**TABLE 8:** Shared pathogenic pathways engaged by environmental neurotoxicants.

Mechanistic pathway	Exemplar exposures	Cellular/Molecular effects	Representative disorders influenced	Refs
Oxidative stress and mitochondrial dysfunction	PM2.5, MeHg, paraquat, rotenone	↑ ROS; complex I inhibition; mtDNA damage; apoptosis	PD, AD, developmental neurotoxicity	Shou et al. (2019)
Neuroinflammation	Air pollution mix, metals, pesticides	Microglial/astrocyte activation; cytokine elevation	PD, AD, MS, cognitive decline	-
Disrupted neurotransmission	Lead, organophosphates, mercury	Ca^2+^ signaling interference; cholinesterase inhibition; GABA/glutamate imbalance	ADHD, PD, seizures, cognitive impairment	-
BBB disruption	PM2.5, metals (Pb, Cd)	Tight junction alteration; increased permeability	Heightened CNS toxin entry; neurodegeneration	-
Epigenetic modifications	Arsenic, BPA, PCBs	DNA methylation/histone modification changes; altered gene expression	Neurodevelopmental disorders; long-latency effects	-

Neuroinflammation occurs when environmental toxins activate microglia and astrocytes, resulting in persistent neuroinflammation (Kwon and Koh, 2020).

Disruption of neurotransmission: Lead hampers calcium signaling; pesticides disrupt dopaminergic transmission; mercury disturbs the balance of glutamatergic and GABA systems. Disruption of the blood–brain barrier (BBB): PM2.5 and heavy metals compromise BBB integrity, enabling neurotoxins to infiltrate the CNS. Epigenetic modifications: Arsenic, BPA, and PCBs influence DNA methylation and histone acetylation in neural cells, impacting long-term gene expression (Qin et al., 2021; Laufer et al., 2022).

### 3.6 Therapeutic and public health implications

Tackling environmental risk factors for neurological disorders involves two main strategies: providing targeted interventions for individuals and enacting broad policy reforms (see Table 9) (Reis et al., 2023).

**TABLE 9:** Multi-level strategies to reduce environmental neurotoxicity and disease risk.

Level	Strategy/Intervention	Expected impact/Evidence	Practical notes	Refs
Individual	Antioxidant- and omega-3–rich diet; adequate selenium	May mitigate pollutant-induced oxidative stress/inflammation	Whole-food emphasis; consider local advisories for fish (Hg)	Calder (2015)
Individual (clinical)	Evaluate exposure history; selective chelation for confirmed heavy-metal toxicity	Chelation can reduce body burden in specific cases; the benefit–to–risk ratio must be weighed	Use evidence-based protocols; monitor minerals/renal function	-
Public health/policy	Air quality improvements; pesticide regulation; EDC phase-outs	Population-level reduction in neurodegenerative disease burden	Requires multisector policy and enforcement	-
Clinical practice	Integrate environmental history into neuro evals; patient counseling	Earlier identification of environmental contributors; targeted prevention	Use standardized questionnaires; local exposure resources	-

Individual-level interventions involve nutritional strategies such as antioxidant-rich diets, omega-3 fatty acids, and selenium, which could help lower oxidative damage caused by pollutants. Although chelation therapy has been explored for heavy metal poisoning, its use remains limited and continues to be debated (Romieu et al., 2008; Smith, 2013).

Public health interventions play a vital role in safeguarding long-term neurohealth by reducing air pollution through clean energy policies, regulating pesticide use, and phasing out harmful endocrine-disrupting chemicals (EDCs) (Khosrorad et al., 2022; Wang et al., 2022; Shekhar et al., 2024; Kassotis et al., 2020).

Clinical considerations: Physicians should integrate environmental history into neurological assessment, particularly in cases of unexplained neurodegeneration or developmental disorders (National Academies Press, 1995).

Ultimately, environmental exposures are preventable factors that contribute to neurological disease burden, and reducing these risks could lead to substantial improvements in population brain health.

## 4 Lifestyle factors and neurological disorders

Lifestyle behaviours (see Table 10), including physical activity, sleep quality, psychosocial stress, and substance use, have significant and cumulative impacts on neurological health. Unlike environmental exposures, these factors are mostly changeable at the individual level, making them key targets for prevention and treatment efforts (Erickson et al., 2011).

**TABLE 10:** Lifestyle Factors and Neurological Disorders; Lifestyle behaviours, including physical activity, sleep quality, psychosocial stress, and substance use, have significant and cumulative impacts on neurological health.

Lifestyle domain	Key effects on neurological health	Core mechanisms	Clinical signals/Evidence	Practical considerations and interventions	Refs
Physical activity	Lower risk of AD, PD, stroke, depression; improved cognition and mood	↑ BDNF → synaptic plasticity and neurogenesis; ↑ cerebral blood flow; ↓ neuroinflammation; ↑ mitochondrial function	Aerobic + resistance training improves cognition in MCI, reduces depressive symptoms, and slows PD progression	Prescribe mixed aerobic/resistance (≥150 min/wk moderate +2×/wk resistance); tailor to ability; monitor adherence and safety	Erickson et al. (2019)
Sleep quality and circadian health	Poor sleep linked to impaired cognition, mood disorders, ↑ AD risk	Deep sleep → glymphatic clearance of Aβ/tau; sleep loss → ↑ oxidative stress, dysregulated pruning, emotional dyscontrol	Treating insomnia and sleep apnea improves cognitive/emotional outcomes	Sleep hygiene; CBT-I; screen and treat OSA (CPAP); regular schedules/light exposure for circadian alignment	Leng et al. (2019)
Psychosocial stress	Chronic stress increases risk for neurodegenerative and psychiatric disorders	HPA overactivation → excess glucocorticoids; ↓ hippocampal neurogenesis/dendrites; microglial activation and ↑ cytokines; epigenetic changes	Stress reduction improves anxiety, depression, and stress-related cognitive deficits	MBSR, yoga, CBT; build social support; address sleep and activity concurrently	McEwen (2017)
Substance use	Alcohol (heavy): cortical atrophy, ↑ dementia; Tobacco: ↑ stroke, cognitive decline; Recreational drugs: memory/executive impairments; Caffeine (moderate): possible neuroprotection	Direct neurotoxicity; thiamine deficiency (alcohol); vascular injury (tobacco); cannabinoid/opioid/monoaminergic pathway disruption; adenosine antagonism (caffeine)	Mixed: light–moderate alcohol (esp. wine) sometimes linked to neuroprotection; high-potency synthetics carry added neuropsychiatric risk	Screen and brief intervention; thiamine for alcohol misuse; smoking cessation; caution with high caffeine (sleep/anxiety); harm-reduction and treatment programs	Topiwala and Ebmeier (2018)
Cross-cutting mechanisms	Behaviors interact to shape resilience vs. vulnerability	Neuroplasticity (BDNF, neurogenesis); inflammation/oxidative stress (cytokines, ROS); energy metabolism (mitochondria); circadian regulation; epigenetic modifications	Multimodal programs show additive benefits across mood, cognition, and function	Combine exercise + sleep optimization + stress management; align with nutrition/pharmacotherapy; tailor to culture, access, and comorbidities	See refs above

### 4.1 Physical activity and neuroplasticity

Regular physical activity is well-known as a key protective factor against neurodegenerative and psychiatric conditions such as migraine (Zhang et al., 2023). Epidemiological research shows that individuals who engage in regular exercise have a reduced risk of developing Alzheimer’s disease, Parkinson’s disease, stroke, and depression (Northey et al., 2018). Importantly, prolonged sedentary behavior appears to confer risk independent of meeting exercise targets, with higher sitting time associated with worse cardiometabolic profiles and increased risk of cognitive decline in older adults (Cai et al., 2023).

Exercise stimulates brain-derived neurotrophic factor (BDNF) signaling, which promotes synaptic plasticity, neurogenesis, and neuronal survival. Specifically, aerobic exercise enhances neurogenesis in the hippocampus and improves memory. Additionally, physical activity increases cerebral blood flow, decreases neuroinflammation, and enhances mitochondrial function (Erickson et al., 2019).

Clinical trials show that structured aerobic and resistance exercises enhance cognitive function in seniors with mild cognitive impairment (MCI), reduce depressive symptoms, and slow the progression of Parkinson’s disease.

### 4.2 Sleep and glymphatic clearance

Sleep is becoming more widely understood as essential for maintaining brain balance. Issues like poor sleep quality, sleep deprivation, or disruptions in circadian rhythm are associated with cognitive decline, mood disorders, and a higher risk of Alzheimer’s disease (Xie et al., 2013).

Circadian misalignment from shift work, chronotype mismatch, and exposure to artificial light at night can disrupt sleep–wake regulation and downstream metabolic and immune rhythms, with growing epidemiological interest in links to cognitive impairment and dementia progression (Filippini et al., 2024).

During deep sleep, the glymphatic system removes metabolic waste products, such as amyloid-β and tau proteins, from the brain. Persistent sleep disruption impairs this cleaning process, resulting in protein accumulation and neurodegeneration (Xie et al., 2013). Moreover, lack of sleep elevates oxidative stress, interferes with synaptic pruning, and impacts emotional regulation (Xie et al., 2013).

Sleep disorders like sleep apnea accelerate neurological decline due to intermittent hypoxia and systemic inflammation. Importantly, treatments such as cognitive-behavioral therapy for insomnia (CBT-I), continuous positive airway pressure (CPAP) for apnea, and circadian rhythm regulation methods have shown benefits in improving cognitive and emotional health (Trauer et al., 2015; Van Der Zweerde et al., 2019).

### 4.3 Psychosocial stress and the hypothalamic pituitary adrenal (HPA) axis

Chronic psychosocial stress is a key contributor to neurodegenerative and psychiatric disorders. Persistent activation of the HPA axis leads to sustained glucocorticoid exposure, which can hinder hippocampal neurogenesis, decrease dendritic complexity, and trigger neuronal apoptosis (McEwen, 2017; Lupien et al., 2009).

Social isolation and loneliness, along with low cognitive stimulation, are increasingly recognized as modifiable contributors to dementia risk–possibly through effects on stress physiology, depression, health behaviors, and reduced cognitive reserve, whereas lifelong learning and mentally stimulating activities may help build reserve and delay clinical expression of pathology (Wang et al., 2023).

Stress-related neuroinflammation, driven by microglial activation and pro-inflammatory cytokines, worsens mood disorders and speeds up neurodegeneration. Epigenetic changes, such as DNA methylation of glucocorticoid receptor genes, could increase vulnerability throughout life (Miller and Raison, 2016).

Protective approaches like mindfulness-based stress reduction (MBSR), yoga, and cognitive-behavioural therapy have proven effective in reducing anxiety, depression, and stress-related cognitive issues (Trauer et al., 2015).

### 4.4 Substance use and neurological risk

Lifestyle also includes patterns of substance use, many of which have direct neurotoxic effects. Dose–response relationships are clinically important: regular moderate caffeine intake is often associated with neutral-to-beneficial cognitive outcomes in observational studies, whereas heavy intake can worsen anxiety, sleep, and blood pressure; for alcohol, heavy use is consistently harmful, and purported benefits of light-to-moderate intake are increasingly questioned due to confounding and genetic (Mendelian randomization) evidence suggesting no truly protective level (Topiwala et al., 2025).

Chronic alcohol consumption can lead to cortical atrophy, thiamine deficiency (resulting in Wernicke–Korsakoff syndrome), increased dementia risk, as well as to ischemic stroke (Mechtcheriakov et al., 2007). Nonetheless, some studies suggest that light to moderate alcohol intake, particularly wine, may offer neuroprotective benefits, largely thanks to polyphenols like resveratrol (Sechi and Serra, 2007).

Nicotine may boost alertness and concentration temporarily; however, long-term tobacco use is strongly linked to higher risks of stroke, cognitive decline, and vascular dementia (Anstey et al., 2007; Zhong et al., 2015).

Chronic use of cannabis, methamphetamine, and opioids can impair memory, executive functions, and emotional regulation. Moreover, highly potent synthetic drugs carry additional neuropsychiatric risks (Meier et al., 2012).

Moderate caffeine intake seems to support brain health, linked to a lower risk of Parkinson’s disease and enhanced cognitive function in older adults. Nonetheless, consuming too much can interfere with sleep and raise anxiety levels (Costa et al., 2010; Ascherio et al., 2001).

### 4.5 Mechanistic insights

Lifestyle behaviors impact neurological health through various interconnected mechanisms. Neuroplasticity: Exercise and stimulating environments boost BDNF levels, support synaptic remodeling, and encourage new neuron growth. Inflammation and oxidative stress: Chronic stress, poor sleep, and substance misuse raise pro-inflammatory cytokines and reactive oxygen species (ROS). Energy metabolism: Physical activity improves mitochondrial function, whereas substance abuse hampers neuronal energy processes. Circadian regulation, including sleep habits and light exposure, influences circadian genes critical for cognition and mood. Epigenetic modifications: Stress and lifestyle choices affect gene expression by changing DNA methylation and histone structures.

### 4.6 Therapeutic implications

Lifestyle modifications are increasingly integrated into neurological prevention and treatment frameworks: Exercise prescriptions are becoming more common as supplementary treatments for depression, dementia, migraine and Parkinson’s disease (Northey et al., 2018).

Sleep hygiene programs, along with circadian interventions, are advised to reduce cognitive decline and stabilize mood (Trauer et al., 2015; Kushida et al., 2012). Stress management techniques such as mindfulness, CBT, and yoga enhance resilience and reduce the likelihood of relapse in psychiatric disorders (Miller and Raison, 2016).

Public health campaigns focused on preventing substance abuse successfully lower the incidence of stroke, dementia, and psychiatric conditions (Zhong et al., 2015). Importantly, lifestyle interventions often work alongside nutritional and pharmacological therapies, providing a comprehensive and cost-effective strategy for brain health that can be utilized across various populations.

## 5 Shared mechanisms Linking nutrition, environment, and Lifestyle

Although nutrition, environmental exposures, and lifestyle habits are frequently examined independently, growing evidence indicates they are deeply interconnected. Their impacts on the central nervous system (CNS) flow through shared biological pathways, which can either increase or decrease the risk of neurological diseases depending on the dominance of protective or harmful factors. Recognizing these standard mechanisms provides a basis for developing comprehensive therapeutic approaches (Feinberg, 2018).

### 5.1 Oxidative stress and mitochondrial dysfunction

Across domains, excess ROS damages lipids, proteins, and mtDNA, impairing ATP production and calcium handling. Environmental pollutants (PM2.5, metals, pesticides) elevate ROS; poor diets and sleep loss compound this. Protective levers include antioxidant-rich dietary patterns (MD/MIND), omega-3s, and mitochondrial support via KD and physical activity (Barnham et al., 2004).

Mitochondria are especially susceptible because of their key functions in energy generation and calcium regulation. Mitochondrial dysfunction is commonly seen in Parkinson’s disease, Alzheimer’s disease, and ALS. Strategies like ketogenic diets, regular exercise, and antioxidant-rich foods support mitochondrial health, underscoring the therapeutic promise of focusing on this shared pathway (McGrattan et al., 2019).

### 5.2 Neuroinflammation and immune dysregulation

Blood–Brain Barrier (BBB) integrity is increasingly recognized as a shared vulnerability point. Diets high in saturated fat and metabolic inflammation can increase BBB permeability, whereas antioxidants and microbiome-derived SCFAs may support tight-junction function; pollutants such as PM2.5 and certain metals can promote endothelial dysfunction and neuroimmune signaling; and lifestyle stressors including sleep deprivation and chronic stress may further amplify BBB disruption and neuroinflammation (Fock and Parnova, 2023).

Circadian rhythm disruption (irregular sleep timing, shift work, and nighttime light exposure) can act as a cross-cutting mechanism by altering glucose and lipid metabolism, inflammatory tone, mitochondrial function, and epigenetic regulation of clock-controlled genes, thereby potentially accelerating neurodegenerative and cerebrovascular processes (Filippini et al., 2024).

Microglia integrate signals from diet (saturated fats vs. polyphenols/omega-3s), pollutants (particulates, pesticides), and stress hormones. Chronic priming promotes cytokines (IL-1β, TNF-α, IL-6) and synaptic dysfunction. Exercise, vitamin D, and polyphenols down-tune pro-inflammatory tone (Heneka et al., 2015).

This indicates that inflammation acts as a crucial connection between nutrition, environment, and lifestyle (Vasefi et al., 2019).

### 5.3 Epigenetic and transcriptomic reprogramming

One-carbon nutrients (folate/B12) govern methyl-donor availability; polyphenols modulate histone acetylation; pollutants (arsenic, BPA, PCBs) and chronic stress reprogram methylation at neurodevelopmental and stress-response loci. These marks help explain long-latency and life-course effects (Feinberg, 2018; Feinberg, 2007).

### 5.4 Gut–brain axis and microbiome modulation

The gut microbiome plays a crucial role in brain health. Factors like diet, environmental toxins, and lifestyle habits affect microbial diversity and metabolism, which in turn influence neurological wellbeing via the gut–brain axis (Fock and Parnova, 2023). Nutritional elements like dietary fiber, polyphenols, and probiotics promote the production of short-chain fatty acids (SCFAs), supporting BBB integrity and decreasing inflammation (Sharon et al., 2019).

In contrast, Western diets, heavy metals, and specific pesticides disturb the gut microbial balance, causing dysbiosis and heightened gut permeability. This situation enables bacterial endotoxins such as lipopolysaccharides to enter the bloodstream, potentially causing neuroinflammation. Stress and disruptions in sleep also modify microbiota composition, strengthening the link between lifestyle and neurological susceptibility.

Microbiome-targeted therapies like prebiotics, probiotics, and fecal microbiota transplantation (FMT) are now under investigation as supplementary treatments for depression, autism, and neurodegenerative diseases.

### 5.5 Vascular and metabolic pathways

Cerebrovascular health serves as a crucial endpoint of integration. Major risk factors for stroke and dementia include conditions like hypertension, diabetes, and obesity, which are mainly influenced by diet and lifestyle as shown in Table 11. Furthermore, environmental pollutants, such as air pollution and tobacco smoke, exacerbate vascular issues by damaging the endothelium and encouraging atherosclerosis (Jiang et al., 2016).

**TABLE 11:** Convergent mechanisms linking modifiable factors to neurological outcomes.

Mechanism	Key drivers (examples)	Protective factors/Interventions	Representative outcomes	Evidence strength	Refs
Oxidative stress and mitochondria	PM2.5, metals (Pb, MeHg), paraquat/rotenone; sleep loss; high-sugar/high-fat diet	MD/MIND; omega-3 (DHA/EPA); KD; physical activity; vitamins C/E, selenium	AD/PD risk, cognitive decline; seizure control (KD)	●●●●●	-
Neuroinflammation	Western diet (saturated fats); pollutants; chronic psychosocial stress	Exercise; vitamin D; polyphenols; omega-3	Depression; MS activity signals; AD progression	●●	-
Epigenetic reprogramming	Folate/B12 deficiency; arsenic/BPA/PCBs; chronic stress	Adequate one-carbon nutrients; polyphenols; stress reduction	Neurodevelopmental disorders; accelerated aging	●●●	-
Gut–brain axis	Western diet; metals/pesticides; sleep disruption	Fiber/fermented foods; pre/probiotics	Mood, cognition; MS signals	●●●	-
Vascular/metabolic	Air pollution; tobacco; inactivity; high sodium/sugar	DASH/MD/MIND; exercise; smoking cessation	Stroke; vascular cognitive impairment	●●●	-

*Grading scheme (e.g., ●●● strong, ●● moderate, ● preliminary).*

Protective dietary patterns like the Mediterranean and DASH, combined with physical activity and stress management, work together to enhance vascular health and glucose metabolism, thereby reducing the risk of both vascular and neurodegenerative diseases.

### 5.6 Systems biology perspective

Considering the intricate relationships between nutrition, environment, and lifestyle, adopting a systems biology approach is essential for fully understanding their impact on neurological health. The use of multi-omics technologies such as genomics, epigenomics, metabolomics, proteomics, and microbiomics, alongside artificial intelligence (AI)- is growing to detect molecular markers linked to environmental and lifestyle factors. These methods will enhance precision medicine by tailoring interventions to each person’s genetic makeup, exposome, and lifestyle profile (Maitre et al., 2022).

### 5.7 Therapeutic integration

The convergence of mechanisms suggests that multi-target approaches, rather than single-drug strategies, might provide more effective treatment results. For example, combining a Mediterranean diet with regular exercise, stress reduction techniques, and minimizing pollutant exposure can collectively help lower the risk of Alzheimer’s disease by addressing inflammation, oxidative stress, and vascular problems simultaneously.

Integrative medicine models, involving nutritionists, neurologists, psychologists, and environmental health specialists, point toward a future of holistic brain healthcare.

## 6 Therapeutic implications

Recognizing that nutrition, environmental exposures, and lifestyle habits influence neurological health has important implications for therapy and public health as shown in Table 12. Unlike genetic predispositions, these factors can be modified, providing opportunities for prevention and disease management. Incorporating these elements into clinical care requires shifting from a predominantly pharmacological approach to a more holistic, patient-centered model.

**TABLE 12:** Inputs–Mechanisms–Outcomes map linking modifiable domains to neurological health.

Inputs	Mechanisms	Outcomes	Refs
Nutrition • Micronutrients (B12, D, Mg) • Diets: MD/MIND, KD • Bioactives: DHA/EPA, polyphenols	Oxidative stress/Mitochondria • Antioxidants; KD ↑ efficiency Neuroinflammation • Omega-3, vitamin D, polyphenols ↓ Epigenetics • One-carbon (folate/B12); polyphenols	Cognition ↑ Neurodegeneration ↓ (signals) Mood stabilization (adjunct)	-
Environment • PM2.5, UFPs, NO2/O3 • Metals: Pb, MeHg, As, Cd • Pesticides; EDCs	Oxidative stress/Mitochondria • ROS; complex I inhibition Neuroinflammation and BBB disruption Epigenetic remodeling	↑ PD/AD risk (assoc.) Cognitive decline Neurodevelopmental effects	-
Lifestyle • Physical activity • Sleep and circadian health • Stress; substances	Neuroinflammation ↓ (exercise) BDNF/Neuroplasticity ↑ Circadian and metabolic alignment Epigenetic impacts (stress)	Lower dementia/stroke risk Improved function/mood Sleep-normalized clearance	-

For clarity, we distinguish (i) primary prevention, reducing risk and delaying onset in healthy or at-risk populations from (ii) adjunctive therapy, supporting standard pharmacological and rehabilitative care after diagnosis. Evidence is generally strongest for prevention and risk reduction, whereas disease modification in advanced disorders remains uncertain.

### 6.1 Nutritional interventions as adjunctive therapies

Dietary modification is one of the most accessible treatment options for neurological disorders. Evidence supports the use of. B vitamins and homocysteine reduction: Taking B12, B6, and folate supplements decreases homocysteine levels and slows brain atrophy in individuals with mild cognitive impairment (Smith et al., 2018).

Clinical limitations should be considered: ketogenic diets may increase cardiometabolic risk in some patients and can lead to micronutrient inadequacies without careful planning; supplements and high-dose antioxidants/polyphenols may interact with medications (e.g., anticoagulants, chemotherapeutics) or blunt training adaptations; and chelation therapy should only be used for confirmed heavy-metal toxicity under strict medical supervision.

Clinical studies show that vitamin D supplementation can lead to improvements in fatigue, motor function, and relapse rates in multiple sclerosis patients (Sintzel et al., 2018). The ketogenic diet, which is a recognized treatment for refractory epilepsy, is currently under investigation for its neuroprotective effects on mitochondrial function in Parkinson’s and Alzheimer’s diseases (Oliveira et al., 2023). Mediterranean and MIND diets: These dietary patterns lower dementia risk and enhance cognitive resilience, likely due to the combined effects of polyphenols, omega-3 fatty acids, and antioxidants. Polyphenol-rich supplements such as curcumin, resveratrol, and flavonoids are currently undergoing clinical trials to evaluate their anti-inflammatory properties and potential to improve cognition (Small et al., 2018).

Personalized nutrition, driven by nutrigenomics and metabolomics, offers the potential to customize dietary guidance based on each person’s unique genetic and metabolic profiles.

### 6.2 Environmental risk reduction

Reducing environmental exposures is a crucial yet often overlooked aspect of treatment. Pollution control efforts that lower exposure to PM2.5 and other air pollutants can reduce the risk of stroke, dementia, and cognitive decline. Implementing policy-level measures, such as switching to clean energy sources and cutting traffic emissions, is crucial (Wang et al., 2022). Heavy metal detoxification involves obtaining safe water, minimizing intake of contaminated food, and, in some instances, employing chelation therapy. Nutritional antioxidants can assist in reducing oxidative stress induced by metals.

Pesticide regulation by limiting workplace exposure, promoting safer alternatives, and monitoring residues in food can help decrease Parkinson’s disease rates. Avoiding endocrine disruptors through public education about BPA-free products, decreasing plastic consumption, and enforcing tighter regulations can help reduce neurodevelopmental risks.

Clinicians can take on a preventive role by including environmental history in neurological assessments.

### 6.3 Lifestyle-based therapies

Lifestyle modification is increasingly recognized as a crucial component in neurological treatment (Trauer et al., 2015; Schenkman et al., 2018). Exercise prescriptions involving structured aerobic and resistance training programs boost cognition in individuals with mild cognitive impairment, reduce depressive symptoms, and improve motor function in Parkinson’s disease.

In addition to physical activity and sleep interventions, cognitive training, structured neurorehabilitation, and social engagement programs can be considered complementary components of multidomain brain-health strategies, particularly for maintaining function and building cognitive reserve.

Sleep interventions like CBT-I, CPAP therapy, and circadian rhythm regulation improve glymphatic clearance and aid in preventing neurodegeneration (Hablitz et al., 2020). Stress management techniques such as mindfulness, yoga, and psychotherapy help reduce neuroinflammation and boost resilience against psychiatric disorders (Alhawat et al., 2024).

Reducing substance use, such as quitting smoking and moderating alcohol consumption, lowers the risk of dementia, stroke, and psychiatric comorbidities. These lifestyle strategies often complement pharmacological treatments, enhancing their effectiveness and reducing side effects (Pan et al., 2019; Osimo et al., 2020).

### 6.4 Multidomain and integrated approaches

Given the link between nutritional, environmental, and lifestyle factors, interventions that target multiple domains can provide greater benefits compared to single-strategy methods (Ngandu et al., 2015).

Sustained adherence is a major barrier: dietary changes, exercise, sleep regularity, and exposure reduction are constrained by socioeconomic status, food environments, occupational schedules, and environmental inequities. Behavioral counseling, community-based programs, and digital support tools (when accessible and culturally adapted) may improve uptake and maintenance (Sindi et al., 2025).

The FINGER trial (Finnish Geriatric Intervention Study to Prevent Cognitive Impairment and Disability) demonstrated that a multidomain program incorporating diet, exercise, cognitive training, and vascular risk monitoring enhances mental performance in at-risk older adults. Similar studies conducted across Europe and Asia also endorse the effectiveness of integrated strategies in postponing dementia onset (Kivipelto et al., 2020).

These findings suggest that upcoming treatments need to be comprehensive, personalized, and multimodal, addressing several pathways simultaneously, including oxidative stress, inflammation, vascular dysfunction, and neuroplasticity.

### 6.5 Implications for precision medicine

Emerging technologies offer tools for tailoring interventions to the individual level. Nutrigenomics involves pinpointing genetic variants, such as APOE4 in Alzheimer’s disease, that influence individual dietary responses (Yassine et al., 2016).

Metabolomics and microbiomics: Profiling metabolic and microbial markers that predict disease risk and response to diet (Lagoumintzis and Patrinos, 2023).

Digital health and wearables: Monitoring sleep, physical activity, stress, and diet in real time to enable adaptive, personalized interventions (Minen and Stieglitz, 2021). Artificial intelligence (AI): Integrating multi-omics and exposome data to predict personalized treatment options (Maitre et al., 2022). Such approaches align with the paradigm of precision neurology, advancing beyond “one-size-fits-all” treatments toward personalized prevention and therapy strategies.

### 6.6 Public health and policy dimensions

Therapeutic implications go beyond the clinic to include population-level interventions. Nutrition policies that promote healthy eating habits, such as reducing trans fats and subsidizing fresh produce (Rushing et al., 2023).

Urban planning strategies to improve air quality and promote active lifestyles.

Workplace wellness initiatives that target stress management, sleep quality, and ergonomic practices (Wang et al., 2022).

Education campaigns highlighting the neurotoxic risks of environmental pollutants and substance abuse (Zhong et al., 2015). By targeting both personal and societal factors, these interventions can significantly reduce the global impact of neurological disorders.

## 7 Future directions

Growing awareness of how nutritional, environmental, and lifestyle factors affect neurological disorders highlights the need for broader, interdisciplinary research. Although some progress has been made, significant gaps still exist in our understanding and in turning these insights into clinical and public health applications. Future multicentre work would benefit from standardised dietary assessment instruments, harmonised exposure metrics (exposomics), and unified neurological/cognitive outcome measures to improve comparability and enable meta-analytic synthesis (Dallere et al., 2025).

### 7.1 Longitudinal and multicentre studies

Most current evidence comes from cross-sectional or short-term studies, which limit the ability to establish causality. To better understand the timing between exposures and neurological outcomes, large-scale, longitudinal, and multicentre research is essential. For example, prospective birth cohort studies examining early-life nutrition and environmental factors could offer insights into the developmental origins of neurological vulnerability. Additionally, long-term intervention trials are vital to confirm the effectiveness of dietary and lifestyle changes in delaying or preventing disease onset.

Implementation science is also needed to bridge the efficacy–effectiveness gap—testing how integrative interventions can be delivered in routine care, adapted to different health systems, and made cost-effective and scalable (Sindi et al., 2025).

### 7.2 Multi-omics and systems biology approaches

Future research should utilize systems biology frameworks that combine genomics, epigenomics, metabolomics, proteomics, and microbiomics. This strategy will enable a thorough understanding of the complex interactions among these fields, helping to clarify how genetic predispositions, environmental factors, and lifestyle choices interact.

Epigenetic mapping could reveal how a lack of nutrients or exposure to toxins changes neural gene expression (Mani et al., 2025).

Microbiome sequencing enhances comprehension of gut–brain interactions in neurodegenerative and psychiatric conditions (Jiang et al., 2025).

Metabolomics can identify circulating biomarkers that can predict dietary responses and the progression of diseases.

When combined with advanced computational tools like artificial intelligence (AI) and machine learning, these methods can improve the predictive modelling of personalized risk and treatment outcomes.

### 7.3 Precision nutrition and lifestyle medicine

Personalized strategies based on genetic and metabolic profiles are usually more effective than general guidelines. For example, people carrying the APOE4 allele may respond differently to dietary fats regarding Alzheimer’s disease risk. Likewise, genetic variations in detoxification pathways can affect susceptibility to environmental toxins (Bermingham et al., 2024).

AI-enabled precision approaches raise practical challenges, including data privacy, algorithmic bias, and unequal access to personalized nutrition, digital health tools, and clean environments; addressing these is essential for ethical and equitable translation (Abrahams and Raimundo, 2025).

Creating precision nutrition and lifestyle medicine frameworks involves combining genetic, clinical, and behavioural data to develop personalized strategies. Wearable devices and digital health platforms provide real-time monitoring of diet, physical activity, sleep, and stress, enabling adaptive interventions.

### 7.4 Novel therapeutic targets

Mechanistic insights into shared pathways highlight new therapeutic strategies: Use mitochondrial regulators such as NAD^+^ enhancers and sirtuin activators to decrease oxidative stress. As NAD^+^ levels decline with age, neuronal redox balance, DNA repair (like PARP), mitophagy, and ATP production are harmed, leading to more oxidative damage (Gore et al., 2015). Nutritional NAD^+^ precursors (such as nicotinamide riboside and nicotinamide mononucleotide) can restore NAD^+^ levels and improve mitochondrial quality control in preclinical models of neurodegeneration; early clinical trials outside neurology show target engagement and safety, supporting further CNS research. Sirtuin activators, including nutraceuticals like resveratrol and advanced small molecules, aim to enhance SIRT1/3 pathways, promoting mitochondrial biogenesis, antioxidant defenses, and synaptic resilience. Together, NAD^+^ boosters and sirtuin activators offer complementary approaches within the mitochondrial–redox pathway (Bonkowski and Sinclair, 2016).

Microbiome-targeted therapies consist of probiotics, prebiotics, and fecal microbiota transplants. The microbiome–gut–brain axis affects neuroimmune function, barrier integrity, and the production of neurotransmitter precursors (Filosa et al., 2018). Meta-analyses of clinical studies indicate that probiotics (and sometimes synbiotics) can help reduce depressive symptoms, likely through mechanisms involving SCFAs, cytokine regulation, and tryptophan metabolism. Prebiotics, however, yield more variable outcomes (Marx et al., 2020). Fecal microbiota transplantation (FMT) is being explored for neurological disorders such as Parkinson’s disease and post-infectious syndromes, with a growing number of case series and early-stage trials (Bruggeman et al., 2024). Nonetheless, large randomized controlled trials and standardized protocols are still required (Chen et al., 2025). Questions about safety, donor screening, and the longevity of benefits are crucial in translating these therapies into widespread use.

Epigenetic therapies, such as dietary methyl donors and drugs that modify epigenetic marks, seek to undo harmful gene regulation changes. Since adverse epigenetic modifications like DNA methylation and histone acetylation build up during injury and inflammation, reversing these is a logical approach to disease treatment. Adequate intake of one-carbon nutrients such as folate, B_12_, and choline that supports the availability of methyl donors and can restore normal neural gene expression in developmental and stress-related models. Pharmacological inhibition of histone deacetylase (HDAC) boosts synaptic plasticity and memory in preclinical neurodegeneration models, and newer brain-penetrant HDAC/DNMT modulators with better selectivity and safety profiles are advancing. Key goals in translation include improving target specificity (e.g., HDAC2 versus HDAC1), determining optimal treatment timing, and combining therapies with rehabilitation and cognitive interventions (Abel and Zukin, 2008).

Neuroimmune modulators target microglial activation caused by environmental and lifestyle stressors. Microglial priming and NLRP3-inflammasome activation link these stressors to synaptic damage and the spread of tau and amyloid. Small-molecule NLRP3 inhibitors, like MCC950 and newer agents that can cross the blood-brain barrier, reduce IL-1β signalling and improve cognition and pathology in AD models (Liang et al., 2022; Johnson et al., 2023). CSF1R inhibitors, which temporarily deplete and then allow microglial repopulation, can reset harmful microglial states and lower amyloid and tau pathology in mice. Precision therapies that modulate, rather than eliminate, microglial phenotypes, along with careful safety monitoring for infection risk and vascular effects, are vital for translating these approaches into clinical practice.

These strategies could improve current pharmacological treatments by emphasizing a multimodal approach to disease modification instead of solely providing symptomatic relief.

### 7.5 Policy and global health perspectives

Future research should extend beyond laboratory and clinical environments to encompass public health and policy domains. Key focus areas include launching nationwide dietary campaigns to lower salt and sugar consumption, offering subsidies for healthier foods, enacting stricter regulations on air pollution, pesticides, and endocrine disruptors, designing urban areas that encourage physical activity, reduce stress, and enhance sleep quality, and increasing access to preventive care, especially in low- and middle-income countries facing a growing prevalence of neurological diseases’ is rising.

Research priorities increasingly emphasize critical windows (prenatal development, adolescence, and ageing) and sex- and gender-specific differences in nutrition, toxin susceptibility, and lifestyle effects, which may inform tailored prevention and precision approaches. These policy initiatives will be essential in addressing the social factors that influence neurological health on a broad scale.

### 7.6 Toward an integrative model of brain health

Ultimately, advancing neurological care relies on adopting an integrative approach that connects nutrition, environment, lifestyle, and pharmacology. This strategy involves interdisciplinary collaboration among neurologists, nutritionists, toxicologists, psychologists, and policy experts. Patient-centered strategies focus on education, empowerment, and self-management of modifiable risk factors. Translational pipelines speed up applying fundamental science discoveries into clinical and community settings. This model aims to improve outcomes for individuals with neurological disorders and reduce the global disease burden through proactive prevention.ion.

## 8 Conclusion

Neurological disorders are complex, multifactorial conditions influenced by genetics and modifiable factors such as diet, environment, and lifestyle. This review emphasizes how these factors play a significant role in brain health, affecting the development and progression of neurodegenerative, neurodevelopmental, and psychiatric disorders.

Finally, the impact of integrative brain-health strategies will depend on equitable access to healthy foods, safe and walkable environments, clean air and water, education, and preventive care. For clinicians, incorporating brief dietary assessment, lifestyle counseling (physical activity, sleep, stress, social connection), and an environmental exposure history into routine neurological care may help operationalize risk reduction and supportive management.

Notably, much of the available human evidence remains associative; rigorous longitudinal designs, causal inference methods, and adequately powered interventions are needed to establish causality and identify who benefits most from specific multidomain strategies.

Nutritional factors such as vitamins, minerals, omega-3 fatty acids, and polyphenols support neuronal health, reduce oxidative stress, and modulate neuroinflammation. Environmental exposures, like air pollution, heavy metals, pesticides, and endocrine disruptors, can cause neurotoxicity by triggering oxidative stress, mitochondrial dysfunction, and epigenetic changes. Lifestyle behaviors including physical activity, sleep quality, psychosocial stress, and substance use also impact neuronal plasticity, metabolic health, and immune responses. Immune responses (Feinberg, 2007).

Importantly, these regions target common mechanistic pathways such as oxidative stress, neuroinflammation, mitochondrial dysfunction, vascular issues, and epigenetic reprogramming. Understanding these interconnected mechanisms informs therapeutic approaches that go beyond medication, emphasizing the importance of multidomain strategies, personalized nutrition, lifestyle modifications, and reducing environmental risks (Barnham et al., 2004; Heneka et al., 2015).

The future of neurological care relies on a comprehensive, patient-centered approach that integrates nutrition, environment, and lifestyle factors into prevention and treatment strategies. Advances in systems biology, multi-omics, and digital health are enabling precision medicine techniques that tailor interventions based on individual genetic, metabolic, and exposome data. Simultaneously, public health policies focused on reducing environmental neurotoxin exposure, promoting healthy diets, and encouraging active lifestyles are vital for tackling the rising global prevalence of neurological conditions (Kassotis et al., 2020).

In summary, integrating nutritional, environmental, and lifestyle factors offers a promising framework for prevention and treatment in neuroscience. This method has the potential to enhance cognitive function, slow neurodegeneration, and boost quality of life across various populations. Future research should focus on long-term, multidisciplinary studies and translational strategies that connect basic science, clinical practice, and public health. This will support the development of a more comprehensive and practical approach to maintaining brain health.

## Glossary

AD: Alzheimer’s disease
ADHD: Attention-deficit/hyperactivity disorder
AI: Artificial intelligence
ALS: Amyotrophic lateral sclerosis
AMPA: α-amino-3-hydroxy-5-methyl-4-isoxazolepropionic acid receptor
APOE4: Apolipoprotein E ε4 allele
ASD: Autism spectrum disorder
ATP: Adenosine triphosphate
BBB: Blood–brain barrier
BDNF: Brain-derived neurotrophic factor
BPA: Bisphenol A
CBT-I: Cognitive behavioral therapy for insomnia
CNS: Central nervous system
CPAP: Continuous positive airway pressure
CSF1R: Colony-stimulating factor 1 receptor
DALYs: Disability-adjusted life years
DASH: Dietary Approaches to Stop Hypertension
DHA: Docosahexaenoic acid
DNA: Deoxyribonucleic acid
DNMT: DNA methyltransferase
EDCs: Endocrine-disrupting chemicals
EPA: Eicosapentaenoic acid
FMT: Fecal microbiota transplantation
GABA: Gamma-aminobutyric acid
GBD: Global Burden of Disease
GPx: Glutathione peroxidase
GST: Glutathione S-transferase
HDAC: Histone deacetylase
HPA: Hypothalamic–pituitary–adrenal (axis)
IL-1β: Interleukin-1 beta
IL-6: Interleukin-6
KD: Ketogenic diet
MCI: Mild cognitive impairment
MD: Mediterranean diet
MeHg: Methylmercury
MIND: Mediterranean–DASH Intervention for Neurodegenerative Delay (MIND diet)
MS: Multiple sclerosis
mtDNA: Mitochondrial DNA
MUFA: Monounsaturated fatty acids
**NAD** ^**+**^: Nicotinamide adenine dinucleotide (oxidized form)
NE: Norepinephrine
NLRP3: NLR family pyrin domain-containing 3 (inflammasome)
NMDA: N-methyl-D-aspartate receptor
**NO**_**2**_: Nitrogen dioxide
OSA: Obstructive sleep apnea
**O**_**3**_: Ozone
PARP: Poly(ADP-ribose) polymerase
PCBs: Polychlorinated biphenyls
PD: Parkinson’s disease
PM2.5: Fine particulate matter ≤2.5 μm
PON1: Paraoxonase 1
POPs: Persistent organic pollutants
PUFA: Polyunsaturated fatty acids
RCTs: Randomized controlled trials
RNA: Ribonucleic acid
ROS: Reactive oxygen species
SCFAs: Short-chain fatty acids
SIRT1/3: Sirtuin 1/Sirtuin 3
TNF-α: Tumor necrosis factor alpha
TrxR: Thioredoxin reductase
UFPs: Ultrafine particles
UFPS: Ultrafine particles (as written in text).
